# Shared environment, shared mechanisms: comparing pathways to mental health outcomes among indigenous youth and youth with other ethnic backgrounds

**DOI:** 10.3389/fpsyg.2026.1824428

**Published:** 2026-07-15

**Authors:** Arina Bukina, Ksenia Eritsyan, Natalia Antonova, Larisa Tsvetkova

**Affiliations:** HSE University, Saint Petersburg, Russia

**Keywords:** anxiety, depression, ethnicity, indigenous, mental health

## Abstract

**Background:**

Numerous studies have shown that indigenous populations experience poorer health outcomes compared to people with other backgrounds. However, the interpretation of these disparities remains challenging due to differences in living conditions and social environments, as well as by potential measurement-related biases. Little is known about whether the underlying mechanisms of mental health outcomes differ between indigenous youth and youth of other ethnic backgrounds.

**Methods:**

Using cross-sectional data from an online survey (*N* = 1,258) of indigenous small-numbered youth and youth from other ethnic backgrounds living in the same social and institutional environment, structural equation modeling was applied to examine how ethnic discrimination, satisfaction with the preservation of an ethnic group’s traditions, finances, and housing are associated with depression (PHQ-9), anxiety (GAD-7) and alcohol use in these populations. *Z*-tests were conducted to compare regression coefficient estimates between the two groups.

**Results:**

The results show that experiences of ethnic discrimination are consistently associated with poorer well-being in both groups, while satisfaction with the preservation of an ethnic group’s traditions is marginally negatively associated only with anxiety among youth of other ethnic backgrounds. Financial satisfaction may serve as a potential protective factor for mental health outcomes across both groups, whereas satisfaction with housing shows no significant associations with any of the outcomes. Alcohol use does not serve as a central mechanism linking material and non-material factors to mental health. *Z*-tests indicate that the patterns of associations are largely similar across groups. The study also provides evidence for the strict invariance of the PHQ-9 scale and the partial strict invariance of the GAD-7 scale across the studied populations.

**Conclusion:**

The study suggests that the mechanisms linking material and non-material factors to mental health outcomes are not specific to ethnic background across small-numbered indigenous youth and populations of other ethnic backgrounds living in the same environment. The findings also highlight the need for tailored interventions that address both material and non-material determinants of mental well-being.

## 1 Introduction

Regions of the North, Siberia and the Far East of Russia are home to many indigenous small-numbered peoples. These groups inhabit the territories of traditional settlement of their ancestors, and some of those preserve traditional lifestyles, economic activities, and crafts. Each group numbers fewer than 50 thousand people and recognizes itself as an independent ethnic community (Federal Law No. 104-FZ, 2000). The history of indigenous peoples in Russia, as well as in many other places around the world, includes a period of socio-economic and cultural challenges, which have led to a demographic decline (Petrov, 2008), so these groups are now under the special protection by Russian officials.

Indigenous populations worldwide tend to have poorer health outcomes compared to non-indigenous groups (Valeggia and Snodgrass, 2015; Anderson et al., 2016; Shahunja et al., 2024). Moreover, indigenous youth are reported to experience higher rates of depression, anxiety, and substance use (Hop Wo et al., 2020). Within certain regions of Russia, similar patterns have been observed, where anxiety levels and suicide rates are reported to be higher among the indigenous peoples (Manchuk et al., 2013; Sumarokov et al., 2014; Bokhan et al., 2017).

However, the dominant narrative portraying indigenous populations as experiencing systematically worse mental health outcomes has recently been questioned, as some studies also point out that there are no significant differences in levels of depression and anxiety between indigenous and non-indigenous populations (Kisely et al., 2017). At the same time, conventional mental health instruments may not function equivalently across these groups and may fail to accurately capture mental health symptoms among indigenous peoples (Kisely et al., 2017). As a result, both the presence and absence of observed group differences may reflect measurement artifacts rather than true variation.

Beyond psychometric concerns, much of the existing literature treats indigenous populations as analytically separate, often focusing on them in isolation from other groups. Review studies identify a wide range of risk and protective factors for mental health within indigenous communities living in geographically or culturally specific and to some extent isolated settings (MacDonald et al., 2013; Young et al., 2017). However, few studies have compared whether such factors operate similarly or differently across indigenous peoples and populations of other ethnic backgrounds (Bals et al., 2010; McNamara et al., 2018), especially in case they are not living isolated but share the same environment. As a result, little is known about whether the mechanisms underlying mental health outcomes are shared or ethnic-specific.

To address this gap, the present study focuses on a set of factors that are frequently discussed in the literature as either protective or harmful influences on mental health. Cultural loss and ethnic discrimination are well-established risk factors for poor mental health among indigenous peoples (King et al., 2009; Faruk and Rosenbaum, 2022; Dutkin, 2013; Kairuz et al., 2021). Cultural loss has also been documented among Indigenous Small-Numbered Peoples of the North, Siberia and the Far East of Russia (Kozhemyakina et al., 2022; Burtseva and Bysyina, 2019). At the same time, some adverse influences may be related not to ethnicity, but to broader territorial and socioeconomic conditions. Residents of the Northern, Siberian and Far Eastern regions of Russia may face challenges that are largely material in nature, including inadequate housing and financial hardship (Schneiderman et al., 2023; Emelyanova and Chapargina, 2020; Minakir et al., 2020), which may in turn contribute to poorer psychological well-being (Ridley et al., 2020; Basit et al., 2025). Also, stressors can drive alcohol consumption (Corbin et al., 2013), which in turn may prospectively increase depressive affect and anxiety (Grazioli et al., 2018; Armeli et al., 2015). This behavioral factor may represent a central mechanism linking these factors to mental health outcomes.

Focused on small-numbered youth and youth of other ethnic backgrounds residing in the North, Siberia and the Far East of Russia the current study addresses the following questions:

- How do perceived ethnic discrimination, satisfaction with the preservation of an ethnic group’s traditions, housing, and financial conditions relate to depression, anxiety, and alcohol use among indigenous small-numbered peoples and youth of other ethnic backgrounds?
- What is the role of alcohol in the relationships between these factors and depression and anxiety for indigenous peoples and youth of other ethnic backgrounds?
- Is there a difference in the effects of material and non-material factors on depression, anxiety, and alcohol use between indigenous small-numbered peoples and youth of other ethnic backgrounds?

## 2 Materials and methods

### 2.1 Data

This study utilizes data from a quantitative cross-sectional online survey conducted in November-December 2020 among adolescents and young people aged 14 to 35 (*N* = 1,258) of Russia which was focused on their health and well-being. The data were collected in the regions of Sakha Republic (Yakutia), Irkutsk Region, Saint Petersburg, Kamchatka Region, Murmansk Region, Krasnoyarsk Region, Tomsk Region, and Leningrad Region.

The sample was recruited through educational institutions and non-governmental organizations. Schools, secondary vocational institutions, and universities with a high representation of indigenous populations of the North, Siberia, and the Far East of Russia were identified in different regions with the assistance of local experts. Regional research coordinators facilitated the selection of institutions and organized data collection. Young people not enrolled in formal education were recruited through non-governmental organizations. Data collection in regional areas was conducted with the assistance of local experts, some of whom were themselves representatives of indigenous communities or had long-standing professional and personal connections with the adolescents involved. This contributed to a culturally sensitive and ethically grounded research process. This study was focused on indigenous young people who live not in isolated settings, but in a shared social and material environment with people of other ethnicities, usually in more urbanized settings, which in western research tradition sometimes referred to as urban indigenous people (King et al., 2009). Informed consent was obtained from all participants through a mandatory confirmation checkbox prior to survey access. Only participants who provided informed consent were included in the study. The study received ethical approval from the Ethics Committee (IRB00011060 Herzen State Pedagogical University of Russia IRB #1). To reduce the potential influence of social desirability bias the study was conducted anonymously, no personal identifiable data was collected and no referral was implemented for the participants based on their answers. Only fully completed questionnaires were retained for analysis. Data quality was assessed through consistency checks of responses to open-ended questions. No participants were excluded following this procedure.

Participants were asked to indicate their ethnic background, choosing between identifying as a member of an indigenous small-numbered peoples of the North, Siberia and the Far East of Russia, as a Russian, or as belonging to another ethnic group. Those who selected the indigenous small-numbered option were then asked to specify their specific ethnic identity, including the option to indicate a mixed ethnic background (e.g., “Even and Russian”). Some participants listed ethnicities that, while indigenous (e.g., “Sakha/Yakut”), are not classified as small-numbered according to the official list of indigenous small-numbered peoples of the North, Siberia and the Far East of Russia (Government of the Russian Federation, 2000). These participants were reclassified as members of other ethnic groups for the purpose of this study. As a result of this classification procedure, participants belonging to indigenous small-numbered populations of the North, Siberia and the Far East of Russia comprise 53.3% (*N* = 671) of the total sample, while participants of other ethnic groups make up 46.7% (*N* = 587) (Supplementary Table 1).

Among 25 indigenous small-numbered ethnicities participating in the study, the Evenki are the most represented, accounting for 26.2% of the total indigenous respondents. This was followed by those identifying as Evens (Lamut) (14.9%), Dolgans (11.2%), a mixed group (10.6%), Koryaks (7.9%), Chukchi (6.1%), Selkup (4.0%), Yukaghir (3.7%) and Nenets (3.4%). Other indigenous groups in the sample account for less than 3% (Supplementary Table 2).

### 2.2 Measures

To assess depression levels, the PHQ-9 scale, adapted for a Russian-speaking sample, was used (Kroenke et al., 2001). It consists of nine items that evaluate the frequency of symptoms over the past 2 weeks. Each item is rated using the following options: “Not at all” (0), “Several days” (1), “More than half the days” (2), “Nearly every day” (3). Total scores range from 0 to 27, indicating the severity of depression symptoms.

To assess anxiety levels, the GAD-7 scale, adapted for a Russian-speaking sample, was used (Spitzer et al., 2006). The frequency of seven anxiety symptoms over the past two weeks was measured using the following options: “Not at all” (0), “Several days” (1), “More than half the days” (2), “Nearly every day” (3). The total GAD-7 score ranges from 0 to 21, reflecting the severity of anxiety symptoms.

The frequency of alcohol consumption was assessed using an item from the multi-language ESPAD questionnaire (European Monitoring Centre for Drugs and Drug Addiction, 2020) used for the Russian-speaking samples. Participants were asked on how many occasions they had consumed alcoholic beverages during the past 30 days. The response categories were “0,” “1–2,” “3–5,” “6–9,” “10–19,” “20–39,” “40 or more.” For analysis, the last three response categories were collapsed into a single category “10 or more.”

To measure satisfaction with living conditions, finances and the preservation of an ethnic group’s traditions participants were asked to rate their satisfaction with those aspects of their lives on a scale from 1 to 5, where 1 indicates “Not satisfied at all” and 5 indicates “Fully satisfied.”

Ethnic discrimination was assessed with the question: “Do you ever experience insult, mistreatment or discrimination because of your ethnicity, or feel subjected to prejudice or oppression due to your ethnic background?” Respondents indicated the frequency of such experiences by selecting from the following options: “Never happens,” “Happens sometimes,” “Happens often.”

### 2.3 Data analysis

In the current study CFA (Confirmatory Factor Analysis) is used to assess the factorial structure of the PHQ-9 and GAD-7 scales and to evaluate whether their measurement properties allow for a meaningful comparison between groups. SEM (Structural Equation Modeling) is used to test hypotheses about direct and indirect effects. For CFA and SEM the estimation method “WLSMV” is used. The analysis is conducted using the “lavaan” and “semTools” packages in R. Since the analysis is based on the already collected data the *a priori* power analysis has not been conducted.

Distributions of key variables and items of scales are provided in Supplementary Tables 3 and 4. Indigenous small-numbered peoples and populations of other ethnic backgrounds of the North, Siberia and the Far East of Russia demonstrate differences in the perceived frequency of ethnic discrimination, alcohol consumption levels, satisfaction with finances, housing, and satisfaction with the preservation of an ethnic group’s traditions (Supplementary Table 3).

Polyserial correlations were calculated for key variables in the data (Supplementary Tables 5–7), and all values are statistically significant for the full sample (Supplementary Table 5), so predictors will be allowed to covary in the structural model. All VIF values are below 3. Polychoric correlations for items of the scales are presented in Supplementary Figures 1–3.

To assess the measurement model, CFA was performed on two correlated factors—depression (PHQ-9) and anxiety (GAD-7) scales—as supported by previous studies (Saunders et al., 2023). The global model fit of the model was evaluated using established thresholds: CFI and TLI ≥ 0.95, SRMR < 0.08, RMSEA < 0.05, while RMSEA ≥ 0.10 indicate poor model fit. Local fit problems were identified via correlations residuals, with absolute values >0.10 flagged as problematic (Kline, 2023). The initial measurement model was rejected due to high RMSEA across all groups (Table 1) and multiple correlations residuals exceeding the recommended threshold (Supplementary Figures 4–6). To improve model fit, a series of modifications were introduced incrementally, guided by theoretical justifications and modification indices that were consistently high across all groups. The modifications included correlations of error terms of items of PHQ-9: 3 and 4; 1 and 2; 1 and 4; 1 and 3. Each of these sequential adjustments improved models fit across all groups (Supplementary Figures 7–9; Supplementary Tables 8–10; Table 1).

**Table 1:** Global fit statistics for CFA models.

Model	*χ* ^2^	d*f*	CFI	TLI	RMSEA (CI 90)	SRMR
Initial measurement model: full sample	1314.39***	103	0.962	0.956	0.097 (0.092–0.101)	0.054
Initial measurement model: indigenous	721.46***	103	0.967	0.962	0.095 (0.088–0.101)	0.053
Initial measurement model: other ethnicity	684.39***	103	0.957	0.950	0.098 (0.091–0.105)	0.059
Modified measurement model: full sample	1038.62***	99	0.970	0.964	0.087 (0.082–0.092)	0.045
Modified measurement model: indigenous	566.62***	99	0.975	0.970	0.084 (0.077–0.091)	0.044
Modified measurement model: other ethnicity	569.33***	99	0.965	0.958	0.090 (0.083–0.097)	0.051

*χ*^2^, Chi-square; d*f*, degrees of freedom; CFI, comparative fit index; TLI, Tucker-Lewis index; RMSEA, root mean square error of approximation; CI, confidence interval; SRMR, standardized root mean square residual. ***p < 0.001.

For the final measurement model, for each factor categorical omega coefficient (ωu-cat) exceeds the recommended threshold of 0.70 (McDonald, 1999), and the measure of convergent validity—the AVE (Average Variance Extracted), exceeds 0.50 (Kline, 2023, p. 239) (Supplementary Table 11).

The final measurement model was tested for measurement invariance between indigenous peoples and populations of other ethnic backgrounds using multigroup CFA to determine whether the factors of the model funсtion similarly for both groups. In the current study, an approach for ordered categorical data was used based on the guidelines code of Svetina et al. (2020), which involves testing for configural invariance, as well as equality of thresholds and loadings across groups. The configural model represents the final measurement model. Then, constraints were imposed sequentially: first—on thresholds and then—on thresholds and factor loadings. Models were compared by changes in global fit statistics and chi-square difference test, with each model being evaluated relative to the less constrained one. According to the results (Table 2), there is no worsening of the global fit statistics in any step of the invariance testing procedure, and all chi-square difference tests are insignificant. This indicates scalar invariance of the final measurement model, permitting comparison of structural paths in the SEM across groups. The PHQ-9 and GAD-7 scales were also tested separately for strict measurement invariance, following the guidelines code outlined by Tse et al. (2023). The results supported strict invariance for PHQ-9 scale, permitting observed mean comparison across groups. For GAD-7 scale, partial strict invariance was established, which supports factor mean comparison (Supplementary Tables 12, 13). No significant group differences were observed in PHQ-9 observed scores and GAD-7 latent means (Supplementary Table 3).

**Table 2:** Measurement invariance.

Model	*χ* ^2^	d*f*	CFI	RMSEA	SRMR	ΔCFI	ΔRMSEA	ΔSRMR	*p* (Δ*χ*^2^)
1	1135.72***	198	0.971	0.087	0.047	—	—	—	—
2	1152.07***	214	0.971	0.084	0.047	0	−0.003	0	0.613
3	1114.11***	228	0.973	0.079	0.047	0.002	−0.005	0	0.259

1 = final measurement model = configural model; 2 = configural model + constraints on equality of thresholds; 3 = configural model + constraints on equality of thresholds, loadings. *χ*^2^, Chi-square; d*f*, degrees of freedom; CFI, comparative fit index; RMSEA, root mean square error of approximation; SRMR, standardized root mean square residual, Δ shows changes in the index (index current − index previous), *p* (Δ*χ*^2^), *p*-value for Chi-square difference test. ***p < 0.001.

**Figure 1:**
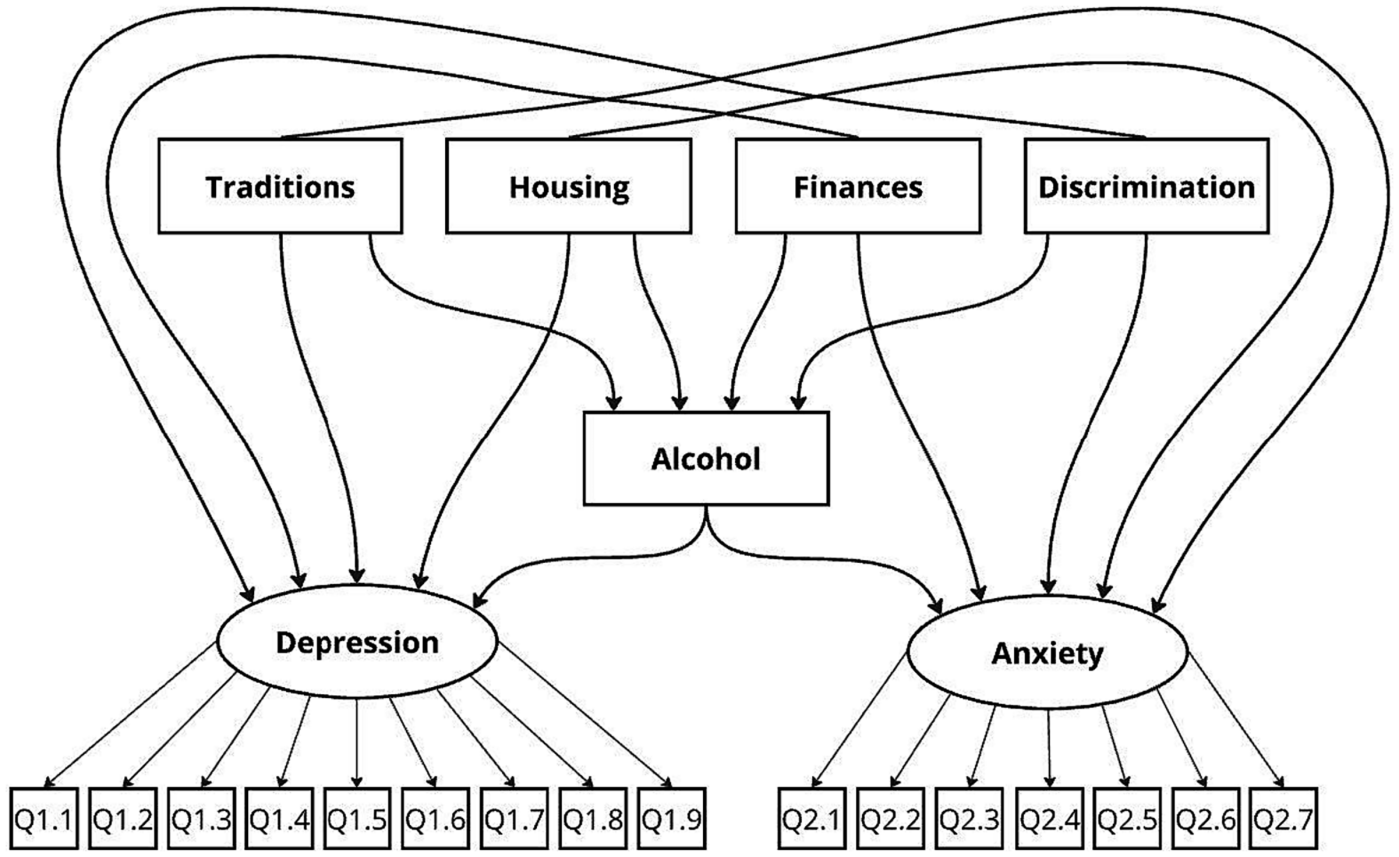
Theoretical model (covariance structure is not specified in the figure).

SEM was used to test the hypotheses of the theoretical model (Figure 1) separately for indigenous small-numbered peoples and populations of other ethnic backgrounds. The global fit statistics for the full model indicate acceptable model fit across all groups (Table 3).

**Table 3:** Global fit statistics for SEM models.

Model	*χ* ^2^	d*f*	CFI	TLI	RMSEA (CI 90)	SRMR
Full model: full sample	1089.08***	169	0.975	0.969	0.066 (0.062–0.070)	0.039
Full model: indigenous	623.34***	169	0.979	0.974	0.063 (0.058–0.069)	0.040
Full model: other ethnicity	636.59***	169	0.971	0.963	0.069 (0.063–0.074)	0.049

*χ*^2^, Chi-square; df, degrees of freedom; CFI, comparative fit index; TLI, Tucker-Lewis index; RMSEA, root mean square error of approximation; CI, confidence interval, SRMR, standardized root mean square residual. ***p < 0.001.

For both groups direct and indirect effects were assessed. Differences between groups in each regression coefficient were tested using z-tests (Paternoster et al., 1998) for the unstandardized direct and indirect estimates. Given the exploratory nature of this part of the analysis, to account for multiple comparisons, *p*-values for *z*-tests were also adjusted using the Benjamini-Hochberg false discovery rate (FDR) correction (Benjamini and Hochberg, 1995).

## 3 Results

Table 4 summarizes the standardized estimates of direct and indirect effects for both groups, along with uncorrected and FDR-corrected *p*-values for *z*-tests.

**Table 4:** Direct and indirect effects for indigenous and other ethnicity groups.

Path	Standardized estimate-indigenous (*p*-value)	Standardized estimate-other ethnicity (*p*-value)	*p* (*z*-tests)	*p*-FDR (*z*-tests)
Traditions → Depression	0.055 (*p* = 0.241)	−0.070 (*p* = 0.204)	0.085	0.598
Traditions → Anxiety	0.064 (*p* = 0.159)	*−0.094 (p = 0.084)*	0.027	0.381
Traditions → Alcohol	−0.001 (*p* = 0.984)	−0.095 (*p* = 0.214)	0.334	0.816
Traditions → Alcohol → Depression	0.000 (p = 0.984)	−0.010 (*p* = 0.295)	0.324	0.797
Traditions → Alcohol → Anxiety	0.000 (p = 0.984)	−0.007 (*p* = 0.403)	0.410	0.797
Discrimination → Depression	**0.237 (*p* = 0.000)**	**0.136 (*p* = 0.027)**	0.151	0.706
Discrimination → Anxiety	**0.217 (p = 0.000)**	**0.129 (*p* = 0.047)**	0.291	0.816
Discrimination → Alcohol	**0.143 (*p* = 0.018)**	0.107 (*p* = 0.101)	0.691	0.855
Discrimination → Alcohol → Depression	0.005 (*p* = 0.532)	0.012 (*p* = 0.183)	0.572	0.797
Discrimination → Alcohol → Anxiety	0.006 (*p* = 0.401)	0.008 (*p* = 0.283)	0.914	0.914
Housing → Depression	0.049 (*p* = 0.450)	−0.022 (*p* = 0.765)	0.458	0.855
Housing → Anxiety	0.022 (*p* = 0.732)	−0.026 (*p* = 0.736)	0.634	0.855
Housing → Alcohol	−0.079 (*p* = 0.276)	−0.107 (*p* = 0.303)	0.829	0.892
Housing → Alcohol → Depression	−0.003 (*p* = 0.576)	−0.012 (*p* = 0.369)	0.511	0.797
Housing → Alcohol → Anxiety	−0.004 (*p* = 0.489)	−0.008 (*p* = 0.437)	0.698	0.797
Finances → Depression	**−0.180 (*p* = 0.005)**	**−0.184 (*p* = 0.007)**	0.898	0.898
Finances → Anxiety	**−0.176 (*p* = 0.004)**	*−0.121 (p = 0.097)*	0.566	0.855
Finances → Alcohol	−0.021 (*p* = 0.770)	−0.081 (*p* = 0.388)	0.615	0.855
Finances → Alcohol → Depression	−0.001 (*p* = 0.790)	−0.009 (*p* = 0.416)	0.429	0.797
Finances → Alcohol → Anxiety	−0.001 (*p* = 0.776)	−0.006 (*p* = 0.455)	0.599	0.797
Alcohol → Depression	0.032 (*p* = 0.525)	**0.111 (*p* = 0.050)**	0.350	0.816
Alcohol → Anxiety	0.045 (*p* = 0.372)	0.071 (*p* = 0.207)	0.733	0.855

The values of significant paths are in bold (*p* ≤ 0.05). The values of paths that are close to statistical significance are in italic (*p* < 0.1). *p* (*z*-tests) = uncorrected *p*-value for *z*-tests for the comparison of unstandardized estimates between groups, *p*-FDR (*z*-tests) = *p*-value for *z*-tests corrected for multiple comparisons using the Benjamini-Hochberg FDR method. *R*^2^ (Indigenous): 0.037 (Alcohol), 0.088 (Depression), 0.083 (Anxiety). *R*^2^ (Other ethnicity): 0.079 (Alcohol), 0.117 (Depression), 0.082 (Anxiety). Full tables with main parameters are provided in Supplementary Tables 14–16.

Satisfaction with the preservation of the ethnic group’s traditions is not significantly related to depression and alcohol use in either indigenous peoples or the group of another ethnic background. In indigenous small-numbered peoples, satisfaction with the preservation of the ethnic group’s traditions is unrelated to anxiety, whereas in the group of another ethnic background, it is negatively associated with anxiety and slightly above the conventional threshold for statistical significance. This effect is the only association that significantly differs between groups before FDR correction, but the difference becomes non-significant after correction for multiple comparisons.

In both groups, experiences of ethnic discrimination are positively associated with symptoms of depression and anxiety. Among indigenous participants, ethnic discrimination is positively associated with alcohol use. No such association is observed in populations of other ethnic backgrounds. However, the magnitude of the effect does not differ significantly between the groups.

Contrary to predictions, satisfaction with housing is not significantly related to depression, anxiety and alcohol consumption in either group. As was expected, satisfaction with finances is negatively associated with depression levels in both groups, with anxiety levels in indigenous population, while among individuals with other ethnic backgrounds, the association with anxiety levels is slightly above the conventional threshold for statistical significance. However, there are no effects of financial satisfaction on alcohol consumption in both groups. *Z*-test for all of these estimates suggests that there are no statistically significant differences between groups in terms of these effects.

Alcohol use is not significantly related to depression and anxiety for indigenous small-numbered peoples, while it is positively associated with depression levels, but is not related to anxiety in the populations of other ethnic backgrounds. In indigenous peoples and populations of other ethnic backgrounds, indirect effects through alcohol consumption are insignificant. Results of z-tests again do not show significant differences in the magnitudes of these direct and indirect effects between groups.

## 4 Discussion

The current study shows that both material and non-material factors are relevant for understanding mental health outcomes of urbanized indigenous peoples and populations of other ethnic backgrounds living in shared environments.

While most research on ethnic discrimination focuses on its effects on minority populations, the experiences of majority groups are often overlooked. In the present study, both indigenous peoples and populations of other ethnic backgrounds reported the experience of ethnic-based discrimination, with the group of another ethnicity facing it to a lesser extent, which is consistent with previous research (Bals et al., 2010). The experience of ethnic discrimination is universally associated with worse mental health outcomes in both indigenous small-numbered peoples and populations of other ethnic backgrounds. Also, in certain regions populations of other ethnic backgrounds may represent a local minority group, which may increase their exposure to discrimination. Ethnic composition in specific locations and institutions could also serve as an important moderator of the relationships between discrimination and mental health (Barry et al., 2024).

The effect of satisfaction with the preservation of the ethnic group’s traditions is observed only in the populations of other ethnic backgrounds, specifically in relation to anxiety. This factor was assumed to predominantly affect the indigenous small-numbered peoples, given the ongoing erosion of their cultural practices (Kozhemyakina et al., 2022; Burtseva and Bysyina, 2019). Instead, it seems that the populations of other ethnic backgrounds may experience a potential protective effect observing their traditions being protected, possibly due to their stronger preservation and widespread practice. Notably, dissatisfaction with the preservation of the ethnic group’s traditions is not exclusive to indigenous populations, as populations of other ethnic backgrounds also show some level of dissatisfaction. Previous studies suggested that engagement in cultural practices can support the well-being of indigenous peoples (MacDonald et al., 2013), though this statement may be expanded to the groups of other ethnic backgrounds and they may also benefit from such engagement. To the best of our knowledge, this potential effect has not been systematically examined in populations within multi-ethnic settings, and it represents an important direction for future research. The current study did not assess the extent of engagement of people in their ethnic group traditions or dimensions of cultural identity and spirituality, which may be relevant to mental wellness (Snowshoe et al., 2017), so further research should therefore adopt more comprehensive measures. This limitation is especially relevant given the age of respondents (14–35). Indigenous youth may feel disconnected from their cultural traditions, as many of these practices began to fade long ago. Moreover, this study was focused on urban indigenous youth who reside in shared territories with other ethnic groups and may adopt elements of their cultural practices, which could further reduce the salience of their cultural identity. While they may feel a sense of loss due to the historical importance of these traditions, their emotional connection may not be as strong, especially if they have not directly engaged in these cultural practices. This could explain why satisfaction with the preservation of the ethnic group’s traditions is not related to their well-being. However, the loss of culture might have a more profound effect on older generations of indigenous peoples who witnessed the process of the disappearance of these traditions and probably were more engaged in this culture. This suggests that further research is needed to explore the generational differences in the impact of cultural loss on well-being.

Among material factors that are not as widely studied as predictors of negative outcomes in similar populations (ethnic groups), satisfaction with housing appears unrelated to the studied outcomes. In contrast, higher satisfaction with finances may serve as a potential protective factor for mental health for both groups. These findings regarding finances align with previous literature (Ridley et al., 2020). The more prominent role of finances in mental health outcomes, compared to housing, may be explained by the broader impact of financial security on overall well-being. It is also important to consider that these factors are interrelated, as satisfaction with housing often depends on financial resources.

This study does not support the notion that alcohol consumption serves as a central mechanism linking the examined stressors to mental health among young people belonging to small-numbered indigenous groups and of other ethnic backgrounds of the North, Siberia and the Far East of Russia. This is evidenced by the absence of indirect effects, which is attributed to the limited number of stressors associated with alcohol consumption and inconsistent associations between alcohol and mental health outcomes across groups. Nonetheless, further research in other populations may reveal stronger or more consistent patterns in these relationships. Low overall rates of alcohol use, observed in this study, may have contributed to the lack of associations. Previous research indicates that associations of alcohol use and mental health outcomes are usually observed in cases of moderate or severe alcohol use (Puddephatt et al., 2022), highlighting the importance of dose–response effects. However, in the current research, only occasions of alcohol consumption were assessed, without information on the beverage type and quantity, which represents another limitation of the research. Also, the relatively young age of the sample, which includes many students, may partly explain the lower prevalence of alcohol use. Limited access to alcohol or underreporting due to social desirability may as well have contributed to this pattern. Prior research also points to the bidirectionality between mental health and substance use (D’Aquino et al., 2026; Baek and Yoon, 2026). In the present study, the direction of effects was set from alcohol use to depression and anxiety based on theoretical framing in which alcohol is conceptualized as a behavioral coping mechanism linking stressors to mental health outcomes. However, future longitudinal studies are needed to test the direction of these associations.

The current study also aimed to examine whether the associations between the factors and mental health outcomes and alcohol use operate similarly across small-numbered indigenous and other populations in case they are living in similar, predominantly urbanized environments. The findings suggest that these effects are largely universal. One potential exception in these terms relates to the possible difference between groups in the effect of satisfaction with the preservation of an ethnic group’s traditions on anxiety. However, the difference appears weak and potentially spurious, so this finding is preliminary and requires further investigation. Also, there are no differences between groups in levels of depression and anxiety. This pattern aligns with some previous research reporting minimal or no differences in mental health outcomes between indigenous peoples and non-indigenous populations in other parts of the world (Kisely et al., 2017). Importantly, the absence of substantial differences in both prevalence and pathways suggests that the indigenous small-numbered peoples may not represent a fundamentally distinct population in terms of mental health outcomes and related mechanisms. The present study focuses on indigenous people who currently reside in shared territories with people of other ethnic backgrounds and, to a larger extent, experience a similar institutional and social environment. Such contextual factors may partly explain the convergence in observed patterns.

Although indigenous small-numbered populations of the North, Siberia and the Far East of Russia may represent a diverse set of ethnic groups, the present study focused on the aggregated category of indigenous small-numbered peoples. This approach was justified by the common legal status of these populations, as well as similar historical trajectories. However, future research based on larger samples may assess within-group variation and determine whether specific cultural or historical factors moderate the pathways associated with mental health outcomes.

The results of this study underscore the value of comparative studies that focus not only on the differences in outcomes, but also on the mechanisms that underlie them. However, valid cross-group comparisons require appropriately validated assessment tools. The current study supports the reliability and validity of the Russian version of the PHQ-9 and GAD-7 scales. The results indicate that these scales are suitable for use in both indigenous small-numbered peoples and other populations of the North, Siberia and the Far East of Russia. The establishment of strict and partial strict invariance of the PHQ-9 and GAD-7 scales, respectively, suggests that these instruments function similarly across indigenous small-numbered peoples and populations of other ethnic backgrounds living within the same social and institutional environment. Notably, no previous studies have examined measurement invariance for these scales across these ethnic groups in Russian samples. While the current study did not include item-level comprehension checks, future studies may additionally benefit from piloting instruments with cognitive interviewing techniques to ensure item-level comprehension equivalence across ethnic groups.

Given the cross-sectional nature of the data, this study cannot establish causal relationships. In addition, although being collected on an anonymous basis, the institutional based recruitment format may affect response patterns, particularly for sensitive topics examined in the study. Future studies should address the limitations of the current research by employing more sophisticated designs, including longitudinal approaches and more nuanced measurement tools, including more rigorous and objective measures of mental health and perception of the preservation of traditions. This would enable a deeper understanding of the mechanisms shaping mental health outcomes among indigenous peoples and populations of other ethnic backgrounds.

Nevertheless, this study raises a number of important questions that warrant further investigations. From a policy perspective, the findings point to several potentially modifiable factors that may serve as useful targets for mental health interventions. These include efforts to reduce ethnic discrimination and to enhance psychosocial protective factors, such as positive ethnic identity, collective self-esteem, and social support, which may buffer the negative effects of experiences of discrimination on mental health (Bardol et al., 2020). Moreover, economic support programs and measures to improve both perceived and actual financial security may have significant benefits for mental health.

## 5 Conclusion

This study examined how material and non-material factors relate to mental health outcomes and alcohol use among small-numbered indigenous peoples and youth with other ethnic backgrounds residing on the same territories. While several hypothesized associations were supported, others were only partially confirmed or not observed. Financial satisfaction consistently emerged as a potential protective factor against depression and anxiety, unlike housing satisfaction. Experiences of ethnic-based discrimination were associated with poorer well-being in both groups. Contrary to expectations, satisfaction with the preservation of an ethnic group’s traditions was unrelated to mental health outcomes in small-numbered indigenous youth. However, for the youth of other ethnic backgrounds this link is negative which could be interpreted as a potential protective effect against anxiety. Alcohol use did not represent a central mechanism linking these stressors to mental health outcomes. The underlying mechanisms shaping mental health outcomes, as well as the overall levels of depression and anxiety, are largely similar across indigenous peoples and populations of other ethnic backgrounds living within the same social and institutional environment. No substantial ethnic-based differences were observed. These findings highlight the need for further research and the importance of addressing both material and non-material factors in mental health interventions.

## Data Availability

The datasets presented in this article are not readily available because of participant consent restrictions. Requests to access the datasets should be directed to KE, ksenia.eritsyan@gmail.com.
