# Supplementary material for: Shared environment, shared mechanisms: comparing pathways to mental health outcomes among indigenous youth and youth with other ethnic backgrounds
Source: Front Psychol. 2026 Jul 15;17:1824428. doi: 10.3389/fpsyg.2026.1824428 (PMC13416346; doi:10.3389/fpsyg.2026.1824428)
Supplement: Supplementary file 1 [file Table_1.DOCX]

***Supplementary Material***

## 1. Data description

### **Supplementary Table 1.** Descriptive characteristics of sample

|  | **Indigenous small numbered**  **(N=671)** | **Other ethnicity**  **(N=587)** | **Overall**  **(N=1258)** |
| --- | --- | --- | --- |
| **Age** |  |  |  |
| Mean (SD) | 22.1 (6.26) | 19.0 (4.90) | 20.6 (5.87) |
| Median [Min, Max] | 21.0 [14.0, 35.0] | 17.0 [14.0, 35.0] | 19.0 [14.0, 35.0] |
| **Gender** |  |  |  |
| Female | 452 (67.4%) | 381 (64.9%) | 833 (66.2%) |
| Male | 219 (32.6%) | 206 (35.1%) | 425 (33.8%) |
| **Type of Place of Residence** |  |  |  |
| Large city / Regional center | 207 (30.8%) | 122 (20.8%) | 329 (26.2%) |
| District center / Small town / Urban-type settlement | 192 (28.6%) | 119 (20.3%) | 311 (24.7%) |
| Village | 269 (40.1%) | 345 (58.8%) | 614 (48.8%) |
| Nomadic housing | 3 (0.4%) | 1 (0.2%) | 4 (0.3%) |

###

### **Supplementary Table 2.** Indigenous small-numbered peoples represented in the sample

|  | **Female**  **(N=452)** | **Male**  **(N=219)** | **Overall**  **(N=671)** |
| --- | --- | --- | --- |
| **Indigenous group** |  |  |  |
| Chukchi | 21 (4.6%) | 20 (9.1%) | 41 (6.1%) |
| Chulym | 2 (0.4%) | 0 (0%) | 2 (0.3%) |
| Dolgan | 64 (14.2%) | 11 (5.0%) | 75 (11.2%) |
| Even (Lamut) | 61 (13.5%) | 39 (17.8%) | 100 (14.9%) |
| Evenki | 118 (26.1%) | 58 (26.5%) | 176 (26.2%) |
| Itelmen | 10 (2.2%) | 4 (1.8%) | 14 (2.1%) |
| Kamchadal | 5 (1.1%) | 5 (2.3%) | 10 (1.5%) |
| Khanty | 9 (2.0%) | 2 (0.9%) | 11 (1.6%) |
| Koryak | 35 (7.7%) | 18 (8.2%) | 53 (7.9%) |
| Mansi | 2 (0.4%) | 1 (0.5%) | 3 (0.4%) |
| Mixed group | 51 (11.3%) | 20 (9.1%) | 71 (10.6%) |
| Nanai | 1 (0.2%) | 0 (0%) | 1 (0.1%) |
| Nenets | 16 (3.5%) | 7 (3.2%) | 23 (3.4%) |
| Nivkhi | 1 (0.2%) | 1 (0.5%) | 2 (0.3%) |
| Sámi | 10 (2.2%) | 4 (1.8%) | 14 (2.1%) |
| Selkup | 18 (4.0%) | 9 (4.1%) | 27 (4.0%) |
| Telengit | 2 (0.4%) | 0 (0%) | 2 (0.3%) |
| Tofalar (Tofa) | 7 (1.5%) | 6 (2.7%) | 13 (1.9%) |
| Udege | 1 (0.2%) | 0 (0%) | 1 (0.1%) |
| Ulchi | 1 (0.2%) | 0 (0%) | 1 (0.1%) |
| Veps | 2 (0.4%) | 1 (0.5%) | 3 (0.4%) |
| Yukaghir | 15 (3.3%) | 10 (4.6%) | 25 (3.7%) |
| Alyutor | 0 (0%) | 1 (0.5%) | 1 (0.1%) |
| Enet | 0 (0%) | 1 (0.5%) | 1 (0.1%) |
| Ket | 0 (0%) | 1 (0.5%) | 1 (0.1%) |

### **Supplementary Table 3.** Descriptive statistics - key variables

|  | **Indigenous**  **(N=671)** | **Other ethnicity**  **(N=587)** | **Overall**  **(N=1258)** | **Difference tests** |
| --- | --- | --- | --- | --- |
| **Discrimination** |  |  |  | Chi-squared test: χ2 = 21.369; p < 0.001;  p (FDR)< 0.001 |
| Never happens | 467 (69.6%) | 475 (80.9%) | 942 (74.9%) |  |
| Happens sometimes | 171 (25.5%) | 93 (15.8%) | 264 (21.0%) |  |
| Happens often | 33 (4.9%) | 19 (3.2%) | 52 (4.1%) |  |
| **Satisfaction with preservation of ethnic group’s traditions** |  |  |  | Chi-squared test: χ2 = 16.201; p = 0.003;  p (FDR) = 0.003 |
| Not satisfied at all | 73 (10.9%) | 52 (8.9%) | 125 (9.9%) |  |
| Slightly satisfied | 117 (17.4%) | 83 (14.1%) | 200 (15.9%) |  |
| Neutral | 220 (32.8%) | 158 (26.9%) | 378 (30.0%) |  |
| Mostly satisfied | 141 (21.0%) | 152 (25.9%) | 293 (23.3%) |  |
| Fully satisfied | 120 (17.9%) | 142 (24.2%) | 262 (20.8%) |  |
| **Satisfaction with housing** |  |  |  | Chi-squared test: χ2 = 42.735; p < 0.001;  p (FDR)< 0.001 |
| Not satisfied at all | 87 (13.0%) | 66 (11.2%) | 153 (12.2%) |  |
| Slightly satisfied | 100 (14.9%) | 53 (9.0%) | 153 (12.2%) |  |
| Neutral | 164 (24.4%) | 105 (17.9%) | 269 (21.4%) |  |
| Mostly satisfied | 162 (24.1%) | 130 (22.1%) | 292 (23.2%) |  |
| Fully satisfied | 158 (23.5%) | 233 (39.7%) | 391 (31.1%) |  |
| **Satisfaction with finances** |  |  |  | Chi-squared test: χ2 = 35.11; p < 0.001;  p (FDR)< 0.001 |
| Not satisfied at all | 112 (16.7%) | 82 (14.0%) | 194 (15.4%) |  |
| Slightly satisfied | 146 (21.8%) | 105 (17.9%) | 251 (20.0%) |  |
| Neutral | 224 (33.4%) | 156 (26.6%) | 380 (30.2%) |  |
| Mostly satisfied | 138 (20.6%) | 142 (24.2%) | 280 (22.3%) |  |
| Fully satisfied | 51 (7.6%) | 102 (17.4%) | 153 (12.2%) |  |
| **Alcohol consumption last 30 days (recoded)** |  |  |  | Chi-squared test: χ2 = 10.262; p = 0.036;  p (FDR) = 0.036 |
| 0 occasions | 469 (69.9%) | 445 (75.8%) | 914 (72.7%) |  |
| 1-2 occasions | 113 (16.8%) | 91 (15.5%) | 204 (16.2%) |  |
| 3-5 occasions | 51 (7.6%) | 24 (4.1%) | 75 (6.0%) |  |
| 6-9 occasions | 13 (1.9%) | 13 (2.2%) | 26 (2.1%) |  |
| 10 or more occasions | 25 (3.7%) | 14 (2.4%) | 39 (3.1%) |  |
| **PHQ-9** |  |  |  | Mann-Whitney U test: W = 193642;  p = 0.608 |
| Mean (SD) | 9.13 (6.24) | 9.39 (6.36) | 9.25 (6.29) |  |
| Median [Min, Max] | 9.00 [0, 27.0] | 9.00 [0, 27.0] | 9.00 [0, 27.0] |  |
| **GAD-7** |  |  |  | Factor mean comparison: p = 0.494(scalar model);  p = 0.227(partial strict model) |
| Mean (SD) | 6.10 (5.39) | 6.38 (5.48) | 6.23 (5.43) |  |
| Median [Min, Max] | 5.00 [0, 21.0] | 6.00 [0, 21.0] | 5.50 [0, 21.0] |  |

###

### **Supplementary Table 4.** Descriptive statistics - PHQ-9 and GAD-7

|  | **Indigenous**  **(N=671)** | **Other ethnicity**  **(N=587)** | **Overall**  **(N=1258)** |
| --- | --- | --- | --- |
| **Q1.1 - PHQ-9.1 Little interest or pleasure in doing things** |  |  |  |
| Not at all | 127 (18.9%) | 83 (14.1%) | 210 (16.7%) |
| Several days | 287 (42.8%) | 272 (46.3%) | 559 (44.4%) |
| More than half the days | 133 (19.8%) | 116 (19.8%) | 249 (19.8%) |
| Nearly every day | 124 (18.5%) | 116 (19.8%) | 240 (19.1%) |
| **Q1.2 - PHQ-9.2 Feeling down, depressed, or hopeless** |  |  |  |
| Not at all | 190 (28.3%) | 167 (28.4%) | 357 (28.4%) |
| Several days | 291 (43.4%) | 249 (42.4%) | 540 (42.9%) |
| More than half the days | 122 (18.2%) | 96 (16.4%) | 218 (17.3%) |
| Nearly every day | 68 (10.1%) | 75 (12.8%) | 143 (11.4%) |
| **Q1.3 - PHQ-9.3 Trouble falling asleep, staying asleep, or sleeping too much** |  |  |  |
| Not at all | 198 (29.5%) | 183 (31.2%) | 381 (30.3%) |
| Several days | 246 (36.7%) | 204 (34.8%) | 450 (35.8%) |
| More than half the days | 123 (18.3%) | 96 (16.4%) | 219 (17.4%) |
| Nearly every day | 104 (15.5%) | 104 (17.7%) | 208 (16.5%) |
| **Q1.4 - PHQ-9.4 Feeling tired or having little energy** |  |  |  |
| Not at all | 161 (24.0%) | 133 (22.7%) | 294 (23.4%) |
| Several days | 265 (39.5%) | 230 (39.2%) | 495 (39.3%) |
| More than half the days | 143 (21.3%) | 123 (21.0%) | 266 (21.1%) |
| Nearly every day | 102 (15.2%) | 101 (17.2%) | 203 (16.1%) |
| **Q1.5 - PHQ-9.5 Poor appetite or overeating** |  |  |  |
| Not at all | 254 (37.9%) | 238 (40.5%) | 492 (39.1%) |
| Several days | 217 (32.3%) | 184 (31.3%) | 401 (31.9%) |
| More than half the days | 119 (17.7%) | 90 (15.3%) | 209 (16.6%) |
| Nearly every day | 81 (12.1%) | 75 (12.8%) | 156 (12.4%) |
| **Q1.6 - PHQ-9.6 Feeling bad about yourself - or that you’re a failure or have let yourself or your family down** |  |  |  |
| Not at all | 285 (42.5%) | 253 (43.1%) | 538 (42.8%) |
| Several days | 217 (32.3%) | 183 (31.2%) | 400 (31.8%) |
| More than half the days | 113 (16.8%) | 93 (15.8%) | 206 (16.4%) |
| Nearly every day | 56 (8.3%) | 58 (9.9%) | 114 (9.1%) |
| **Q1.7 - PHQ-9.7 Trouble concentrating on things, such as reading the newspaper or watching television** |  |  |  |
| Not at all | 258 (38.5%) | 218 (37.1%) | 476 (37.8%) |
| Several days | 267 (39.8%) | 226 (38.5%) | 493 (39.2%) |
| More than half the days | 91 (13.6%) | 84 (14.3%) | 175 (13.9%) |
| Nearly every day | 55 (8.2%) | 59 (10.1%) | 114 (9.1%) |
| **Q1.8 - PHQ-9.8 Moving or speaking so slowly that other people could have noticed. Or, the opposite - being so fidgety or restless that you have been moving around a lot more than usual** |  |  |  |
| Not at all | 384 (57.2%) | 324 (55.2%) | 708 (56.3%) |
| Several days | 155 (23.1%) | 139 (23.7%) | 294 (23.4%) |
| More than half the days | 94 (14.0%) | 95 (16.2%) | 189 (15.0%) |
| Nearly every day | 38 (5.7%) | 29 (4.9%) | 67 (5.3%) |
| **Q1.9 - PHQ-9.9 Thoughts that you would be better off dead or of hurting yourself in some way** |  |  |  |
| Not at all | 413 (61.6%) | 355 (60.5%) | 768 (61.0%) |
| Several days | 135 (20.1%) | 120 (20.4%) | 255 (20.3%) |
| More than half the days | 86 (12.8%) | 66 (11.2%) | 152 (12.1%) |
| Nearly every day | 37 (5.5%) | 46 (7.8%) | 83 (6.6%) |
| **Q2.1 - GAD-7.1 Feeling nervous, anxious or on edge** |  |  |  |
| Not at all | 239 (35.6%) | 204 (34.8%) | 443 (35.2%) |
| Several days | 248 (37.0%) | 230 (39.2%) | 478 (38.0%) |
| More than half the days | 127 (18.9%) | 92 (15.7%) | 219 (17.4%) |
| Nearly every day | 57 (8.5%) | 61 (10.4%) | 118 (9.4%) |
| **Q2.2 - GAD-7.2 Not being able to stop or control worrying** |  |  |  |
| Not at all | 349 (52.0%) | 298 (50.8%) | 647 (51.4%) |
| Several days | 204 (30.4%) | 187 (31.9%) | 391 (31.1%) |
| More than half the days | 88 (13.1%) | 65 (11.1%) | 153 (12.2%) |
| Nearly every day | 30 (4.5%) | 37 (6.3%) | 67 (5.3%) |
| **Q2.3 - GAD-7.3 Worrying too much about different things** |  |  |  |
| Not at all | 268 (39.9%) | 220 (37.5%) | 488 (38.8%) |
| Several days | 236 (35.2%) | 217 (37.0%) | 453 (36.0%) |
| More than half the days | 110 (16.4%) | 93 (15.8%) | 203 (16.1%) |
| Nearly every day | 57 (8.5%) | 57 (9.7%) | 114 (9.1%) |
| **Q2.4 - GAD-7.4 Trouble relaxing** |  |  |  |
| Not at all | 288 (42.9%) | 229 (39.0%) | 517 (41.1%) |
| Several days | 229 (34.1%) | 206 (35.1%) | 435 (34.6%) |
| More than half the days | 104 (15.5%) | 96 (16.4%) | 200 (15.9%) |
| Nearly every day | 50 (7.5%) | 56 (9.5%) | 106 (8.4%) |
| **Q2.5 - GAD-7.5 Being so restless that it is hard to sit still** |  |  |  |
| Not at all | 348 (51.9%) | 304 (51.8%) | 652 (51.8%) |
| Several days | 200 (29.8%) | 172 (29.3%) | 372 (29.6%) |
| More than half the days | 93 (13.9%) | 80 (13.6%) | 173 (13.8%) |
| Nearly every day | 30 (4.5%) | 31 (5.3%) | 61 (4.8%) |
| **Q2.6 - GAD-7.6 Becoming easily annoyed or irritable** |  |  |  |
| Not at all | 231 (34.4%) | 196 (33.4%) | 427 (33.9%) |
| Several days | 255 (38.0%) | 211 (35.9%) | 466 (37.0%) |
| More than half the days | 106 (15.8%) | 102 (17.4%) | 208 (16.5%) |
| Nearly every day | 79 (11.8%) | 78 (13.3%) | 157 (12.5%) |
| **Q2.7 - GAD-7.7 Feeling afraid as if something awful might happen** |  |  |  |
| Not at all | 318 (47.4%) | 276 (47.0%) | 594 (47.2%) |
| Several days | 203 (30.3%) | 172 (29.3%) | 375 (29.8%) |
| More than half the days | 97 (14.5%) | 81 (13.8%) | 178 (14.1%) |
| Nearly every day | 53 (7.9%) | 58 (9.9%) | 111 (8.8%) |

##

## 2. Correlations

### **Supplementary Table 5.** Polyserial correlations - key variables: full sample

|  | **1** | **2** | **3** | **4** | **5** | **6** | **7** |
| --- | --- | --- | --- | --- | --- | --- | --- |
| **1. Alcohol** | - | 0.099*** | 0.086** | 0.136*** | -0.113*** | -0.151*** | -0.126*** |
| **2. Depression** | 0.099*** | - | 0.762*** | 0.193*** | -0.107*** | -0.138*** | -0.19*** |
| **3. Anxiety** | 0.086** | 0.762*** | - | 0.178*** | -0.105*** | -0.131*** | -0.167*** |
| **4. Discrimination** | 0.136*** | 0.193*** | 0.178*** | - | -0.137*** | -0.184*** | -0.135*** |
| **5. Traditions** | -0.113*** | -0.107*** | -0.105*** | -0.137*** | - | 0.506*** | 0.491*** |
| **6. Housing** | -0.151*** | -0.138*** | -0.131*** | -0.184*** | 0.506*** | - | 0.662*** |
| **7. Finances** | -0.126*** | -0.19*** | -0.167*** | -0.135*** | 0.491*** | 0.662*** | - |
| **Note**: ***p ≤ 0.001, **p ≤ 0.01, *p ≤ 0.05. | | | | | | | |

### **Supplementary Table 6.** Polyserial correlations - key variables: indigenous subsample

|  | **1** | **2** | **3** | **4** | **5** | **6** | **7** |
| --- | --- | --- | --- | --- | --- | --- | --- |
| **1. Alcohol** | - | 0.073 | 0.081* | 0.135** | -0.053 | -0.118** | -0.071 |
| **2. Depression** | 0.073 | - | 0.767*** | 0.231*** | -0.047 | -0.089* | -0.155*** |
| **3. Anxiety** | 0.081* | 0.767*** | - | 0.214*** | -0.049 | -0.103* | -0.162*** |
| **4. Discrimination** | 0.135** | 0.231*** | 0.214*** | - | -0.14*** | -0.194*** | -0.174*** |
| **5. Traditions** | -0.053 | -0.047 | -0.049 | -0.14*** | - | 0.502*** | 0.489*** |
| **6. Housing** | -0.118** | -0.089* | -0.103* | -0.194*** | 0.502*** | - | 0.652*** |
| **7. Finances** | -0.071 | -0.155*** | -0.162*** | -0.174*** | 0.489*** | 0.652*** | - |
| **Note**: ***p ≤ 0.001, **p ≤ 0.01, *p ≤ 0.05. | | | | | | | |

###

### **Supplementary Table 7.** Polyserial correlations - key variables: other ethnicity subsample

|  | **1** | **2** | **3** | **4** | **5** | **6** | **7** |
| --- | --- | --- | --- | --- | --- | --- | --- |
| **1. Alcohol** | - | 0.141*** | 0.098* | 0.118* | -0.176*** | -0.175*** | -0.176*** |
| **2. Depression** | 0.141*** | - | 0.755*** | 0.153** | -0.181*** | -0.203*** | -0.235*** |
| **3. Anxiety** | 0.098* | 0.755*** | - | 0.142* | -0.176*** | -0.174*** | -0.185*** |
| **4. Discrimination** | 0.118* | 0.153** | 0.142* | - | -0.109* | -0.136** | -0.057 |
| **5. Traditions** | -0.176*** | -0.181*** | -0.176*** | -0.109* | - | 0.5*** | 0.479*** |
| **6. Housing** | -0.175*** | -0.203*** | -0.174*** | -0.136** | 0.5*** | - | 0.659*** |
| **7. Finances** | -0.176*** | -0.235*** | -0.185*** | -0.057 | 0.479*** | 0.659*** | - |
| **Note**: ***p ≤ 0.001, **p ≤ 0.01, *p ≤ 0.05. | | | | | | | |

### **Supplementary Fig. 1.** Polychoric correlations - PHQ-9 and GAD-7 items: full sample

###
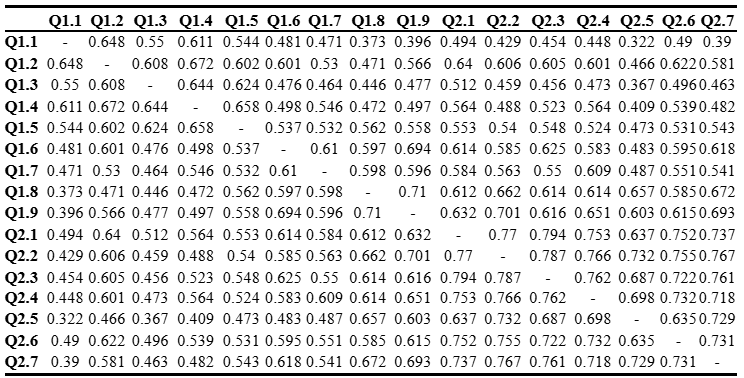


### **Supplementary Fig. 2.** Polychoric correlations - PHQ-9 and GAD-7 items: indigenous subsample

###
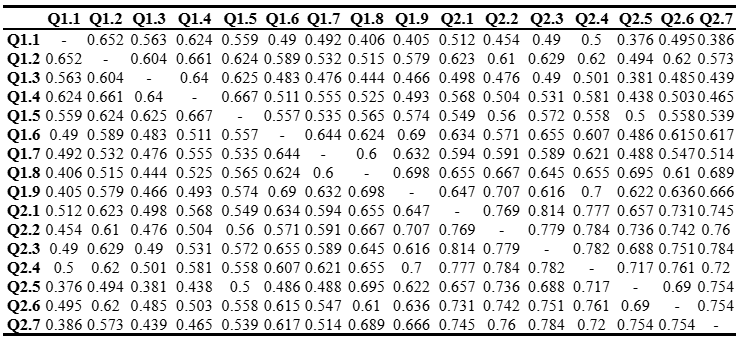


### **Supplementary Fig. 3.** Polychoric correlations - PHQ-9 and GAD-7 items: other ethnicity subsample


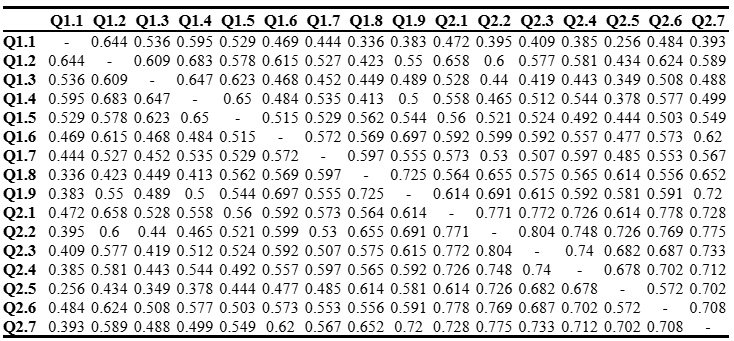


## 3. Correlations residuals for models

### **Supplementary Fig. 4.** Correlations residuals for: Initial measurement model - Full sample


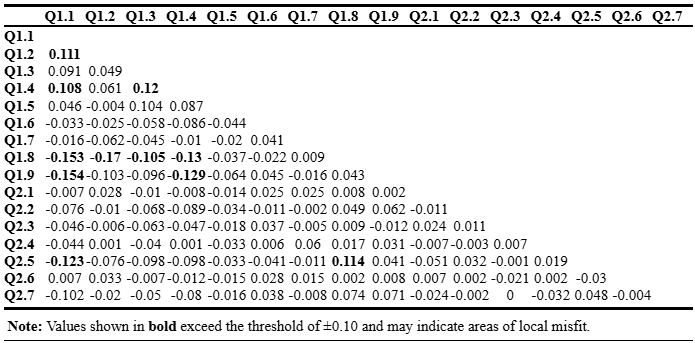


###

### **Supplementary Fig. 5.** Correlations residuals for: Initial measurement model - Indigenous


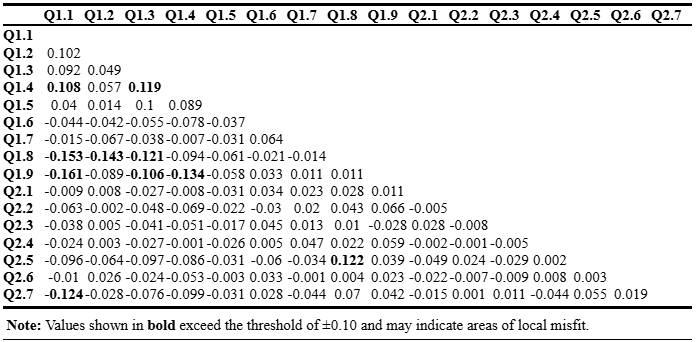


###

### **Supplementary Fig. 6.** Correlations residuals for: Initial measurement model - Other ethnicity


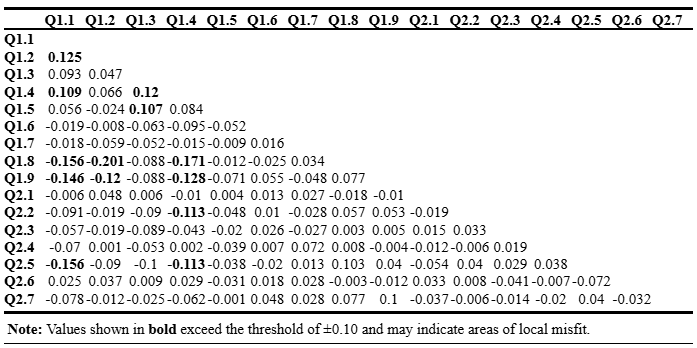


###

### **Supplementary Fig. 7.** Correlations residuals for: Modified measurement model - Full sample


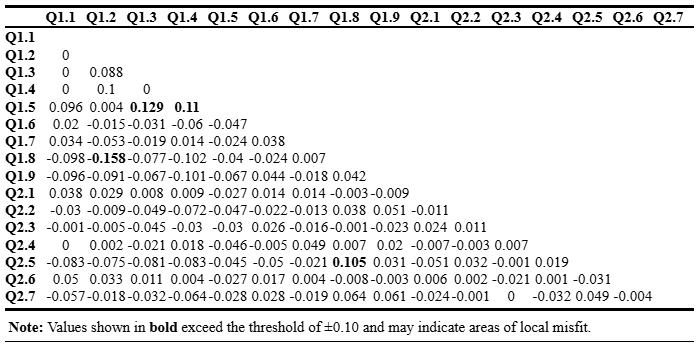


###

### **Supplementary Fig. 8.** Correlations residuals for: Modified measurement model - Indigenous

###
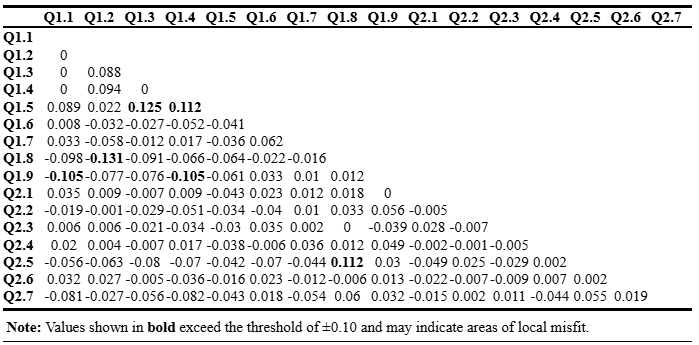


### **Supplementary Fig. 9.** Correlations residuals for: Modified measurement model - Other ethnicity


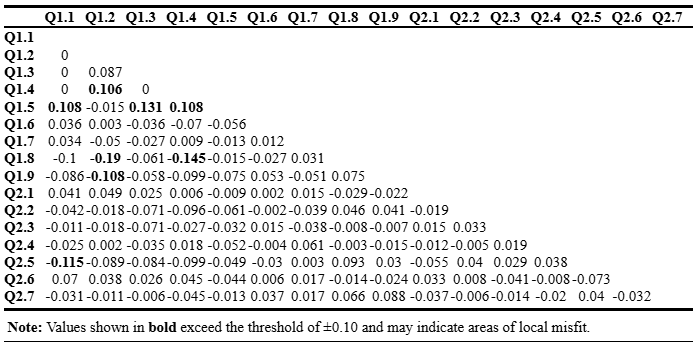


###

## 4. CFA model modifications

###

### Supplementary Table 8. CFA model modifications: full sample

| **Model** | **CFI** | **TLI** | **RMSEA**  **(CI 90)** | **SRMR** | **χ2** | **df** | **P (Δ χ2)** |
| --- | --- | --- | --- | --- | --- | --- | --- |
| **0** | 0.962 | 0.956 | 0.097  (0.092 - 0.101) | 0.054 | 1314.39*** | 103 | - |
| **1** | 0.964 | 0.958 | 0.094  (0.090 - 0.099) | 0.052 | 1240.68*** | 102 | < 0.001 |
| **2** | 0.966 | 0.960 | 0.092  (0.088 - 0.097) | 0.050 | 1180.74*** | 101 | < 0.001 |
| **3** | 0.969 | 0.962 | 0.089  (0.084 - 0.094) | 0.047 | 1099.94*** | 100 | < 0.001 |
| **4** | 0.970 | 0.964 | 0.087  (0.082 - 0.092) | 0.045 | 1038.62*** | 99 | < 0.001 |
| **Note:**  0 = Initial measurement model;  1 = Initial measurement model + Q1.3 ~~ Q1.4 = Model 1;  2 = Model 1 + Q1.1 ~~ Q1.2 = Model 2;  3 = Model 2 + Q1.1 ~~ Q1.4 = Model 3;  4 = Model 3 + Q1.1 ~~ Q1.3 = Model 4.  χ2 = Chi-square, df = degrees of freedom, CFI = Comparative Fit Index, TLI = Tucker-Lewis Index, RMSEA = Root Mean Square Error of Approximation, CI = Confidence Interval, SRMR = Standardized Root Mean Square Residual, P (Δ χ2) = p-value for Chi-square difference test.  ***p < 0.001 | | | | | | | |

###

### **Supplementary Table 9.** CFA model modifications: indigenous subsample

| **Model** | **CFI** | **TLI** | **RMSEA**  **(CI 90)** | **SRMR** | **χ2** | **df** | **P (Δ χ2)** |
| --- | --- | --- | --- | --- | --- | --- | --- |
| **0** | 0.967 | 0.962 | 0.095  (0.088 - 0.101) | 0.053 | 721.46*** | 103 | - |
| **1** | 0.969 | 0.964 | 0.092  (0.086 - 0.099) | 0.051 | 682.25*** | 102 | < 0.001 |
| **2** | 0.971 | 0.965 | 0.090  (0.084 - 0.097) | 0.049 | 651.84*** | 101 | < 0.001 |
| **3** | 0.973 | 0.968 | 0.087  (0.080 - 0.094) | 0.047 | 605.86*** | 100 | < 0.001 |
| **4** | 0.975 | 0.970 | 0.084  (0.077 - 0.091) | 0.044 | 566.62*** | 99 | < 0.001 |
| **Note:**  0 = Initial measurement model;  1 = Initial measurement model + Q1.3 ~~ Q1.4 = Model 1;  2 = Model 1 + Q1.1 ~~ Q1.2 = Model 2;  3 = Model 2 + Q1.1 ~~ Q1.4 = Model 3;  4 = Model 3 + Q1.1 ~~ Q1.3 = Model 4.  χ2 = Chi-square, df = degrees of freedom, CFI = Comparative Fit Index, TLI = Tucker-Lewis Index, RMSEA = Root Mean Square Error of Approximation, CI = Confidence Interval, SRMR = Standardized Root Mean Square Residual, P (Δ χ2) = p-value for Chi-square difference test.  ***p < 0.001 | | | | | | | |

### **Supplementary Table 10.** CFA model modifications: other ethnicity subsample

| **Model** | **CFI** | **TLI** | **RMSEA**  **(CI 90)** | **SRMR** | **χ2** | **df** | **P (Δ χ2)** |
| --- | --- | --- | --- | --- | --- | --- | --- |
| **0** | 0.957 | 0.950 | 0.098  (0.091 - 0.105) | 0.059 | 684.39*** | 103 | - |
| **1** | 0.959 | 0.952 | 0.096  (0.089 - 0.103) | 0.058 | 653.77*** | 102 | < 0.001 |
| **2** | 0.961 | 0.954 | 0.094  (0.087 - 0.101) | 0.055 | 624.24*** | 101 | < 0.001 |
| **3** | 0.963 | 0.956 | 0.092  (0.085 - 0.099) | 0.053 | 591.99*** | 100 | < 0.001 |
| **4** | 0.965 | 0.958 | 0.090  (0.083 - 0.097) | 0.051 | 569.33*** | 99 | < 0.001 |
| **Note:**  0 = Initial measurement model;  1 = Initial measurement model + Q1.3 ~~ Q1.4 = Model 1;  2 = Model 1 + Q1.1 ~~ Q1.2 = Model 2;  3 = Model 2 + Q1.1 ~~ Q1.4 = Model 3;  4 = Model 3 + Q1.1 ~~ Q1.3 = Model 4.  χ2 = Chi-square, df = degrees of freedom, CFI = Comparative Fit Index, TLI = Tucker-Lewis Index, RMSEA = Root Mean Square Error of Approximation, CI = Confidence Interval, SRMR = Standardized Root Mean Square Residual, P (Δ χ2) = p-value for Chi-square difference test.  ***p < 0.001 | | | | | | | |

## 5. Reliability and validity of PHQ-9 and GAD-7

### **Supplementary Table 11.** Reliability and validity of factors

| **Model** | **ωu-cat Depression** | **ωu-cat**  **Anxiety** | **AVE  Depression** | **AVE  Anxiety** |
| --- | --- | --- | --- | --- |
| Modified measurement model - Full sample | 0.860 | 0.927 | 0.550 | 0.732 |
| Modified measurement model - Indigenous | 0.864 | 0.930 | 0.559 | 0.744 |
| Modified measurement model - Other ethnicity | 0.856 | 0.924 | 0.539 | 0.720 |
| **Note:** ωu-cat = categorical omega coefficient, AVE = Average Variance Extracted. | | | | |

## 6. Measurement invariance: PHQ-9 and GAD-7

### Supplementary Table 12. Measurement invariance - GAD-7

| **Model** | **χ2** | **df** | **CFI** | **RMSEA** | **SRMR** | **ΔCFI** | **ΔRMSEA** | **ΔSRMR** | **P**  **(Δχ2)** |
| --- | --- | --- | --- | --- | --- | --- | --- | --- | --- |
| **1** | 142.930*** | 28 | 0.995 | 0.081 | 0.022 | - | - | - | - |
| **2** | 139.265*** | 34 | 0.995 | 0.070 | 0.022 | 0 | -0.011 | 0 | 0.740 |
| **3** | 120.398*** | 47 | 0.997 | 0.050 | 0.022 | 0.002 | -0.020 | 0 | 0.745 |
| **4** | 120.562*** | 52 | 0.997 | 0.046 | 0.023 | 0 | -0.004 | 0.001 | 0.112 |
| **Note:**  Q2.2 - marker variable.  1 = Configural model = GAD-7 scale (fixed loading for Q2.2; fixed factor mean of reference group to 0; fixed latent factor variance to 1 in the reference group; fixed first threshold per item across groups; fixed first and second threshold for Q2.2; fixed unique factor variances to 1 in the reference group);  2 = Metric model = Configural model + fixed loadings across groups;  3 = Scalar model = Metric model + fixed thresholds across groups;  4 = Partial strict model = Scalar model + fixed unique factor variances (variances of Q2.4 and Q2.2 are freely estimated).  χ2 = Chi-square, df = degrees of freedom, CFI = Comparative Fit Index, RMSEA = Root Mean Square Error of Approximation, SRMR = Standardized Root Mean Square Residual, Δ shows changes in the index (Index current - Index previous), P(Δχ2) = p-value for chi-square difference test. ***p < 0.001 | | | | | | | | | |

### Supplementary Table 13. Measurement invariance - PHQ-9

| **Model** | **χ2** | **df** | **CFI** | **RMSEA** | **SRMR** | **ΔCFI** | **ΔRMSEA** | **ΔSRMR** | **P**  **(Δχ2)** |
| --- | --- | --- | --- | --- | --- | --- | --- | --- | --- |
| **1** | 605.499*** | 54 | 0.953 | 0.128 | 0.068 | - | - | - | - |
| **2** | 612.006*** | 62 | 0.953 | 0.119 | 0.068 | 0 | -0.009 | 0 | 0.108 |
| **3** | 548.411*** | 79 | 0.960 | 0.097 | 0.068 | 0.007 | -0.022 | 0 | 0.878 |
| **4** | 494.441*** | 88 | 0.965 | 0.086 | 0.068 | 0.005 | -0.011 | 0 | 0.830 |
| **Note:**  Q1.2 - marker variable.  1 = Configural model = PHQ-9 scale (fixed loading for Q1.2; fixed factor mean of reference group to 0; fixed latent factor variance to 1 in the reference group; fixed first threshold per item across groups; fixed first and second threshold for Q1.2; fixed unique factor variances to 1 in the reference group);  2 = Metric model = Configural model + fixed loadings across groups;  3 = Scalar model = Metric model + fixed thresholds across groups;  4 = Strict model = Scalar model + fixed unique factor variances.  χ2 = Chi-square, df = degrees of freedom, CFI = Comparative Fit Index, RMSEA = Root Mean Square Error of Approximation, SRMR = Standardized Root Mean Square Residual, Δ shows changes in the index (Index current - Index previous), P(Δχ2) = p-value for chi-square difference test. ***p < 0.001 | | | | | | | | | |

## 7. Full tables for effects

### Supplementary Table 14. Direct and indirect effects for indigenous group

| **Path** | **Unstandardized Estimate** | **SE** | **P** | **CI Lower** | **CI Upper** | **Standardized Estimate** |  |
| --- | --- | --- | --- | --- | --- | --- | --- |
| Traditions → Depression | 0.034 | 0.029 | 0.241 | -0.023 | 0.090 | 0.055 |  |
| Housing → Depression | 0.030 | 0.039 | 0.450 | -0.047 | 0.107 | 0.049 |  |
| **Finances → Depression** | **-0.110** | **0.039** | **0.005** | **-0.186** | **-0.034** | **-0.180** |  |
| **Discrimination → Depression** | **0.145** | **0.034** | **0.000** | **0.079** | **0.211** | **0.237** |  |
| Alcohol → Depression | 0.020 | 0.031 | 0.525 | -0.041 | 0.080 | 0.032 |  |
| Traditions → Anxiety | 0.056 | 0.040 | 0.159 | -0.022 | 0.134 | 0.064 |  |
| Housing → Anxiety | 0.019 | 0.056 | 0.732 | -0.091 | 0.129 | 0.022 |  |
| **Finances → Anxiety** | **-0.154** | **0.054** | **0.004** | **-0.260** | **-0.049** | **-0.176** |  |
| **Discrimination → Anxiety** | **0.191** | **0.047** | **0.000** | **0.098** | **0.284** | **0.217** |  |
| Alcohol → Anxiety | 0.039 | 0.043 | 0.372 | -0.046 | 0.124 | 0.045 |  |
| Traditions → Alcohol | -0.001 | 0.060 | 0.984 | -0.119 | 0.116 | -0.001 |  |
| Housing → Alcohol | -0.081 | 0.074 | 0.276 | -0.225 | 0.064 | -0.079 |  |
| Finances → Alcohol | -0.022 | 0.074 | 0.770 | -0.166 | 0.123 | -0.021 |  |
| **Discrimination → Alcohol** | **0.145** | **0.061** | **0.018** | **0.025** | **0.265** | **0.143** |  |
| Traditions → Alcohol→ Depression | 0.000 | 0.001 | 0.984 | -0.002 | 0.002 | 0.000 |  |
| Housing → Alcohol→ Depression | -0.002 | 0.003 | 0.576 | -0.007 | 0.004 | -0.003 |  |
| Finances → Alcohol→ Depression | 0.000 | 0.002 | 0.790 | -0.004 | 0.003 | -0.001 |  |
| Discrimination → Alcohol → Depression | 0.003 | 0.005 | 0.532 | -0.006 | 0.012 | 0.005 |  |
| Traditions → Alcohol→ Anxiety | 0.000 | 0.002 | 0.984 | -0.005 | 0.005 | 0.000 |  |
| Housing → Alcohol → Anxiety | -0.003 | 0.005 | 0.489 | -0.012 | 0.006 | -0.004 |  |
| Finances → Alcohol→ Anxiety | -0.001 | 0.003 | 0.776 | -0.007 | 0.005 | -0.001 |  |
| Discrimination → Alcohol→ Anxiety | 0.006 | 0.007 | 0.401 | -0.007 | 0.019 | 0.006 |  |
| **Note:** SE = Standard Error, P = p-value, CI = Confidence Interval. The lines with significant paths (p ≤ 0.05) are in bold.  R² (Indigenous): 0.037 (Alcohol) , 0.088 (Depression), 0.083 (Anxiety). | | | | | | | |

### Supplementary Table 15. Direct and indirect effects for other ethnicity group

| **Path** | **Unstandardized Estimate** | **SE** | **P** | **CI Lower** | **CI Upper** | **Standardized Estimate** |
| --- | --- | --- | --- | --- | --- | --- |
| Traditions → Depression | -0.039 | 0.031 | 0.204 | -0.100 | 0.021 | -0.070 |
| Housing → Depression | -0.012 | 0.041 | 0.765 | -0.092 | 0.067 | -0.022 |
| **Finances → Depression** | **-0.103** | **0.038** | **0.007** | **-0.177** | **-0.029** | **-0.184** |
| **Discrimination → Depression** | **0.076** | **0.034** | **0.027** | **0.009** | **0.143** | **0.136** |
| **Alcohol → Depression** | **0.061** | **0.031** | **0.050** | **0.000** | **0.121** | **0.111** |
| *Traditions → Anxiety* | *-0.082* | *0.048* | *0.084* | *-0.175* | *0.011* | *-0.094* |
| Housing → Anxiety | -0.023 | 0.068 | 0.736 | -0.157 | 0.111 | -0.026 |
| *Finances → Anxiety* | *-0.106* | *0.064* | *0.097* | *-0.231* | *0.019* | *-0.121* |
| **Discrimination → Anxiety** | **0.113** | **0.057** | **0.047** | **0.002** | **0.224** | **0.129** |
| Alcohol → Anxiety | 0.061 | 0.048 | 0.207 | -0.034 | 0.156 | 0.071 |
| Traditions → Alcohol | -0.096 | 0.078 | 0.214 | -0.248 | 0.056 | -0.095 |
| Housing → Alcohol | -0.109 | 0.106 | 0.303 | -0.316 | 0.098 | -0.107 |
| Finances → Alcohol | -0.083 | 0.096 | 0.388 | -0.271 | 0.106 | -0.081 |
| Discrimination → Alcohol | 0.109 | 0.067 | 0.101 | -0.021 | 0.239 | 0.107 |
| Traditions → Alcohol→ Depression | -0.006 | 0.006 | 0.295 | -0.017 | 0.005 | -0.010 |
| Housing → Alcohol→ Depression | -0.007 | 0.007 | 0.369 | -0.021 | 0.008 | -0.012 |
| Finances → Alcohol→ Depression | -0.005 | 0.006 | 0.416 | -0.017 | 0.007 | -0.009 |
| Discrimination → Alcohol → Depression | 0.007 | 0.005 | 0.183 | -0.003 | 0.016 | 0.012 |
| Traditions → Alcohol→ Anxiety | -0.006 | 0.007 | 0.403 | -0.020 | 0.008 | -0.007 |
| Housing → Alcohol → Anxiety | -0.007 | 0.009 | 0.437 | -0.023 | 0.010 | -0.008 |
| Finances → Alcohol→ Anxiety | -0.005 | 0.007 | 0.455 | -0.018 | 0.008 | -0.006 |
| Discrimination → Alcohol→ Anxiety | 0.007 | 0.006 | 0.283 | -0.005 | 0.019 | 0.008 |
| **Note:** SE = Standard Error, P = p-value, CI = Confidence Interval. The lines with significant paths (p ≤ 0.05) are in bold. The lines with paths that are close to statistical significance (p < 0.1) are in italic.  R² (Other ethnicity): 0.079 (Alcohol), 0.117 (Depression), 0.082 (Anxiety). | | | | | | |

### Supplementary Table 16. Comparison of unstandardized estimates between groups

| **Path** | **Unstandardized Estimate (Indigenous)** | **Unstandardized Estimate**  **(Other ethnicity)** | **Z** | **P** | **P**  **(FDR)** |
| --- | --- | --- | --- | --- | --- |
| Traditions → Depression | 0.034 | -0.039 | 1.720 | 0.085 | 0.598 |
| Housing → Depression | 0.030 | -0.012 | 0.742 | 0.458 | 0.855 |
| Finances → Depression | -0.110 | -0.103 | -0.129 | 0.898 | 0.898 |
| Discrimination → Depression | 0.145 | 0.076 | 1.435 | 0.151 | 0.706 |
| Alcohol → Depression | 0.020 | 0.061 | -0.935 | 0.350 | 0.816 |
| **Traditions → Anxiety** | **0.056** | **-0.082** | **2.209** | **0.027** | 0.381 |
| Housing → Anxiety | 0.019 | -0.023 | 0.477 | 0.634 | 0.855 |
| Finances → Anxiety | -0.154 | -0.106 | -0.573 | 0.566 | 0.855 |
| Discrimination → Anxiety | 0.191 | 0.113 | 1.056 | 0.291 | 0.816 |
| Alcohol → Anxiety | 0.039 | 0.061 | -0.341 | 0.733 | 0.855 |
| Traditions → Alcohol | -0.001 | -0.096 | 0.965 | 0.334 | 0.816 |
| Housing → Alcohol | -0.081 | -0.109 | 0.217 | 0.829 | 0.892 |
| Finances → Alcohol | -0.022 | -0.083 | 0.503 | 0.615 | 0.855 |
| Discrimination → Alcohol | 0.145 | 0.109 | 0.397 | 0.691 | 0.855 |
| Traditions → Alcohol→ Depression | 0.000 | -0.006 | 0.986 | 0.324 | 0.797 |
| Housing → Alcohol→ Depression | -0.002 | -0.007 | 0.657 | 0.511 | 0.797 |
| Finances → Alcohol→ Depression | 0.000 | -0.005 | 0.791 | 0.429 | 0.797 |
| Discrimination → Alcohol → Depression | 0.003 | 0.007 | -0.566 | 0.572 | 0.797 |
| Traditions → Alcohol→ Anxiety | 0.000 | -0.006 | 0.824 | 0.410 | 0.797 |
| Housing → Alcohol → Anxiety | -0.003 | -0.007 | 0.389 | 0.698 | 0.797 |
| Finances → Alcohol→ Anxiety | -0.001 | -0.005 | 0.525 | 0.599 | 0.797 |
| Discrimination → Alcohol→ Anxiety | 0.006 | 0.007 | -0.108 | 0.914 | 0.914 |
| **Note:** Z = z-value, P = uncorrected p-value for z-tests for the comparison of **unstandardized** estimates between groups, P (FDR) = p-value corrected for multiple comparisons using the Benjamini-Hochberg FDR method (applied separately for direct and indirect effects). The lines with paths that may significantly differ between groups are in bold. | | | | | |
